# Heterojunctions of p-BiOI Nanosheets/n-TiO_2_ Nanofibers: Preparation and Enhanced Visible-Light Photocatalytic Activity

**DOI:** 10.3390/ma9020090

**Published:** 2016-01-30

**Authors:** Kexin Wang, Changlu Shao, Xinghua Li, Fujun Miao, Na Lu, Yichun Liu

**Affiliations:** Center for Advanced Optoelectronic Functional Materials Research, and Key Laboratory of UV-Emitting Materials and Technology, Northeast Normal University, Ministry of Education, 5268 Renmin Street, Changchun 130024, China; wangkx217@nenu.edu.cn (K.W.); lixh781@nenu.edu.cn (X.L.); miaofj888@nenu.edu.cn (F.M.); lun242@nenu.edu.cn (N.L.)

**Keywords:** BiOI/TiO_2_, p-n heterojunction, nanofiber, photocatalysis

## Abstract

p-BiOI nanosheets/n-TiO_2_ nanofibers (p-BiOI/n-TiO_2_ NFs) have been facilely prepared via the electrospinning technique combining successive ionic layer adsorption and reaction (SILAR). Dense BiOI nanosheets with good crystalline and width about 500 nm were uniformly assembled on TiO_2_ nanofibers at room temperature. The amount of the heterojunctions and the specific surface area were well controlled by adjusting the SILAR cycles. Due to the synergistic effect of p-n heterojunctions and high specific surface area, the obtained p-BiOI/n-TiO_2_ NFs exhibited enhanced visible-light photocatalytic activity. Moreover, the p-BiOI/n-TiO_2_ NFs heterojunctions could be easily recycled without decreasing the photocatalytic activity owing to their one-dimensional nanofibrous structure. Based on the above, the heterojunctions of p-BiOI/n-TiO_2_ NFs may be promising visible-light-driven photocatalysts for converting solar energy to chemical energy in environment remediation.

## 1. Introduction

Semiconductor photocatalysts have been promising media to degrade pollutants through converting solar energy to chemical energy [1]. In recent years, bismuth oxyhalides BiOX (X = Cl, Br, I), as p-type semiconductors, have attracted considerable attentions owing to their unique layered crystal structure and indirect optical transition characteristic. The layer structure consists of (Bi_2_O_2_)^2+^ layers interleaved by double halogen atoms layers, which can present an internal static electric field and induce the separation of photogenerated carries more efficiently. Furthermore, the indirect transition band gap is necessary for electrons to be emitted to valence band by a certain k-space distance, which reduces the recombination probability of photoelectrons and holes [2,3]. These advantages lead to remarkable photocatalytic activities in degradation of organic compounds [4,5,6,7]. Notably, the p type BiOI (p-BiOI) nanostructures have the narrowest band gap (~1.8 eV) among BiOX (X = Cl, Br, I) nanostructures. Therefore, the p-BiOI nanostructures are considered excellent visible light photocatalysts that can utilize more solar light energy in photocatalysis. Nowadays, many efforts have been employed to improve the photocatalytic efficiency of p-BiOI nanostructures [8,9]. Constructing p-n heterojunctions is considered an effective method to improve the separation efficiency of photogenerated carries due to their strong internal electric field [10,11,12,13]. Many kinds of p-n heterojunctions based on p-BiOI, such as BiOI/ZnTiO_3_ [14], BiOI/Zn_2_SnO_4_ [15], BiOI/ZnO [16], BiOI/Bi_4_Ti_3_O_12_ [17], *etc.*, have been reported with increased photocatalytic activity.

Among many n type semiconductors, Titanium dioxide (TiO_2_) nanostructures have been widely studied as good photocatalysts due to their high efficiency, chemical stability, nontoxicity, low cost, *etc.* [18,19,20,21,22,23,24]. Coupling p-BiOI nanostructures with n-TiO_2_ nanostructures to form p-BiOI/n-TiO_2_ heterojunctions would hinder the recombination of photogenerated carries more effectively. To date, p-BiOI/n-TiO_2_ nanoparticles have been widely reported with enhanced visible-light photocatalytic activity [25,26,27]. However, the suspended nanoparticles tend to aggregate during the synthesis process and be lost in the separation and recycling process, resulting in a reduction of specific surface area and photocatalytic performance. Compared with nanoparticles, one-dimensional nanofibers with a high surface-to-volume ratio are more favorable for both photocatalytic activity and recycling characteristics [28,29]. In fact, our group has previously constructed heterojunctions of p-BiOCl nanosheets/n-TiO_2_ nanofibers [30] and p-MoO_3_ nanosheets/n-TiO_2_ nanofibers [31], both of which show enhanced ultraviolet photocatalytic activities and recycling properties. Therefore, there is interest in constructing p-BiOI/n-TiO_2_ heterojunctions using electrospun TiO_2_ nanofibers as n type semiconductor because of the following advantages: (1) besides the internal electric field of the p-n heterojunction, the one-dimensional characters of TiO_2_ nanofibers could act as charge transfer channels facilitating higher charge separation efficiencies; (2) the three-dimensional open structure and large specific surface area of TiO_2_ nanofibers provide more active sites for the assembly of secondary nanostructures with high densities; and (3) their nanofibrous nonwoven web structure can be easily separated from fluid by sedimentation.

Taking the above factors into account, in this work, the p-type BiOI nanosheets were successfully synthesized on n-type electrospun TiO_2_ nanofibers by successive ionic layer adsorption and reaction (SILAR) at room temperature. The contents of BiOI in the heterojunctions of p-BiOI nanosheets/n-TiO_2_ nanofibers (p-BiOI/n-TiO_2_ NFs) could be well controlled by adjusting the cycles of SILAR. X-ray photoelectron spectra showed that both Ti 2p peaks of p-BiOI/n-TiO_2_ NFs shifted to higher binding energies than that of TiO_2_ nanofibers, suggesting effective electrons transfer from TiO_2_ to BiOI in the formation of p-n heterojunction. The p-BiOI/n-TiO_2_ NFs exhibited favorable visible-light photocatalytic activity for degradation of methyl orange (MO), which can be ascribed to the high specific surface area and the as-formed p-n heterojunctions. Moreover, the heterojunctions of p-BiOI/n-TiO_2_ NFs could be well recycled by sedimentation without decreasing their photocatalytic activities.

## 2. Results and Discussion

### 2.1. Morphologies

**Figure 1:**
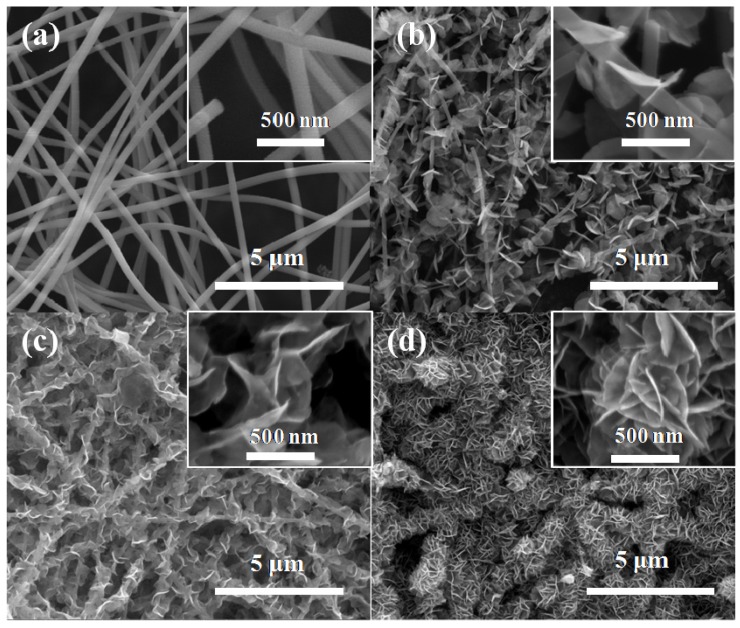
(**a**) SEM images of TiO_2_ nanofibers; (**b**) BiOI/TiO_2_-C10; (**c**) BiOI/TiO_2_-C20; and (**d**) BiOI/TiO_2_-C30 at low magnification and high magnification (insets).

Figure 1 shows the scanning electron microscopy (SEM) images of different samples. It can be seen that TiO_2_ nanofibers with a diameter about 250 nm are relative smooth without secondary structures. After SILAR process, as observed in Figure 1b–d, the TiO_2_ nanofibers become rough and are decorated with ultrathin BiOI nanosheets and remain as one-dimension nanofibers structure. When the cycles of SILAR process increase to 30 for the BiOI/TiO_2_-C30, the amount of the BiOI nanosheets increases significantly compared with those of BiOI/TiO_2_-C10 and BiOI/TiO_2_-C20. These results suggest that the heterojunctions of p-BiOI/n-TiO_2_ NFs may have a higher specific surface area, which is good for the photocatalytic reactions.

**Figure 2:**
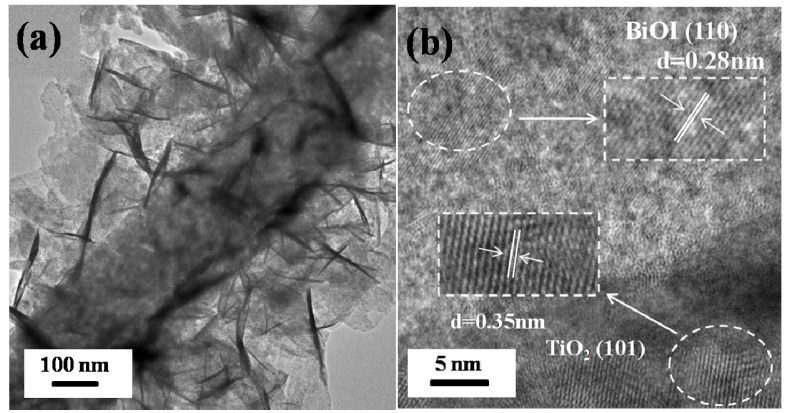
(**a**) TEM; and (**b**) HRTEM images of BiOI/TiO_2_-C30.

Figure 2a,b shows the typical TEM images of BiOI/TiO_2_-C30. It can be observed that numerous BiOI nanosheets are randomly distributed on TiO_2_ nanofibers. The BiOI nanosheets are very thin, which coincides with the results of SEM observations. The high-resolution transmission electron microscopy (HRTEM) image of the heterojunctions displays two types of lattice fringes, as shown in Figure 2b. One set of the fringes spacing is *ca.* 0.35 nm, corresponding to the (101) plane of the anatase crystal structure of TiO_2_. Another set of the fringes spacing measures *ca.* 0.28 nm, which corresponds to the (110) lattice spacing of the BiOI. It indicates that heterojunctions are composed of TiO_2_ nanofibers and BiOI nanosheets with exposed {001} facets. The exposed {001} facets may have excellent photocatalytic activity for BiOI under visible-light irradiation as reported [32]. Therefore, the surface reactivity may also be improved by decorating TiO_2_ nanofibers with BiOI nanosheets.

### 2.2. Structure Characterization

**Figure 3:**
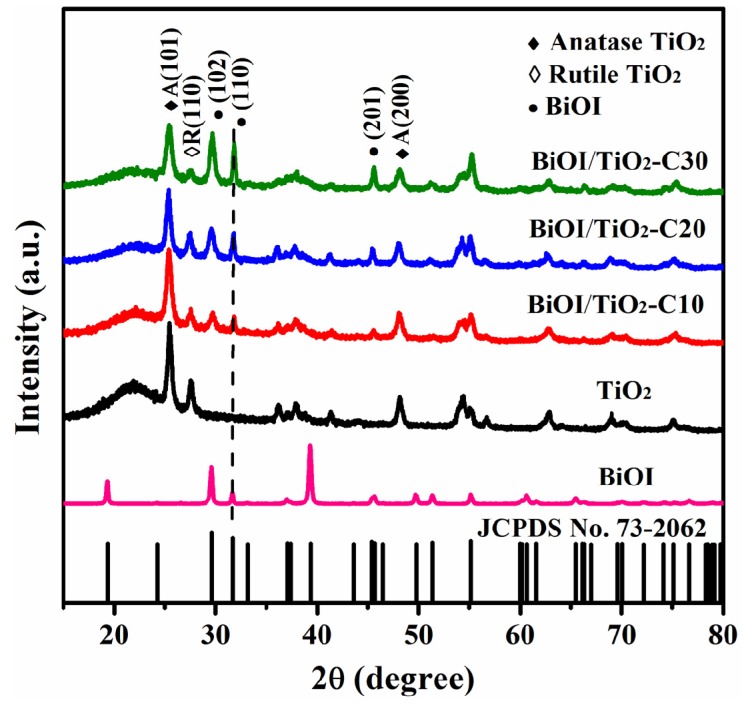
XRD patterns of different samples.

Figure 3 shows the X-ray diffraction (XRD) patterns of pure TiO_2_ nanofibers, p-BiOI/n-TiO_2_ NFs and BiOI nanosheets. For TiO_2_ nanofibers, all peaks are attributed to the anatase of TiO_2_ (JCPDS No. 21-1272) and the rutile of TiO_2_ (JCPDS No. 21-1276). For p-BiOI/n-TiO_2_ NFs, besides the characteristic peaks of TiO_2_ (solid and hollow diamonds), there are some new strong patterns that can be indexed as tetragonal phase of BiOI (JCPDS No. 73-2062). The diffraction peaks of BiOI (solid circles) are gradually intensified as the SILAR cycles increased from 0 to 30, as shown in Figure 3. No other characteristic peaks of impurities are observed. In particular, the domination of (110) plane in the pattern suggests that the exposed facets of BiOI nanosheets are mainly {001}. This result is consistent with SEM and TEM analyses.

### 2.3. Composition and Chemical States

**Figure 4:**
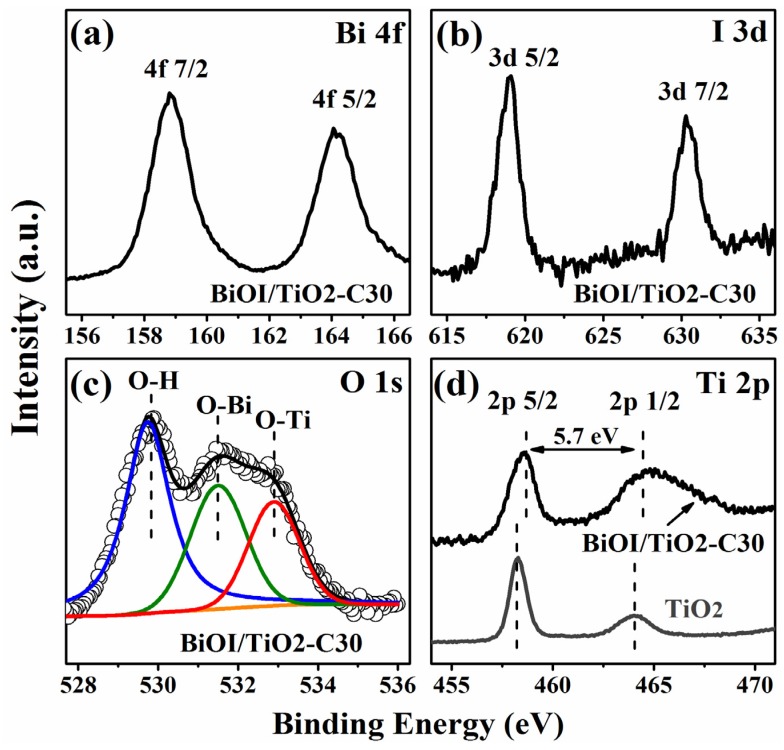
(**a**) XPS spectra of Bi 4f; (**b**) I 3d; and (**c**) O 1s for BiOI/TiO_2_-C30; (**d**) XPS spectra of Ti 2p for TiO_2_ nanofibers (bottom) and BiOI/TiO_2_-C30 (top).

X-ray photoelectron spectroscopy (XPS) measurements are also performed to further investigate the chemical compositions and chemical states of p-BiOI/n-TiO_2_ NFs. The typical high resolution XPS spectrum of Bi 4f is shown in Figure 4a. The peaks at 158.82 eV and 164.14 eV correspond to Bi 4f_3/2_ and Bi 4f_1/2_, respectively, which indicates a normal state of Bi^3+^ in BiOI/TiO_2_-C30 [33]. Figure 4b reveals the high-resolution XPS spectrum of I 3d. The two peaks at 630.3 eV and 618.8 eV are attributed to I 3d_3/2_ and I 3d_5/2_, respectively, which indicates that the chemical state of iodine is I^−1^ in BiOI/TiO_2_-C30 [34]. The deconvolution of the O 1s spectrum in Figure 4c implies that more than one chemical state of O 1s exists in the BiOI/TiO_2_-C30. The peaks with lower binding energies at 529.8 eV and 531.5 eV correspond to the stronger Bi-O and Ti-O bond, respectively. The higher bonding energy of 532.9 eV might be caused by adsorbed water and surface hydroxyl groups (O_OH_), which may also lead to an enhanced photocatalytic property [35]. The splitting between Ti 2p_1/2_ and Ti 2p_3/2_ are both 5.7 eV for TiO_2_ and BiOI/TiO_2_-C30, suggesting a normal state of Ti^4+^ in pure TiO_2_ nanofibers and BiOI/TiO_2_-C30 [36,37]. However, for BiOI/TiO_2_-C30, the binding energy of Ti 2p_3/2_ locates at 458.7 eV, which is about 0.4 eV higher than that of pure TiO_2_ nanofibers (458.3 eV). This can be explained as follow: when p type BiOI nanosheets are deposited on n type TiO_2_ nanofibers, the electrons in TiO_2_ nanofibers would diffuse to BiOI, forming p-n heterojunctions; thus, in the space charge region, TiO_2_ is positively charged which could increase the binding energy of electrons in Ti 2p chemical states. Similar results have been observed in the heterojunctions of p-MoO_3_ nanosheets/n-TiO_2_ nanofibers [26].

### 2.4. Nitrogen Adsorption

**Figure 5:**
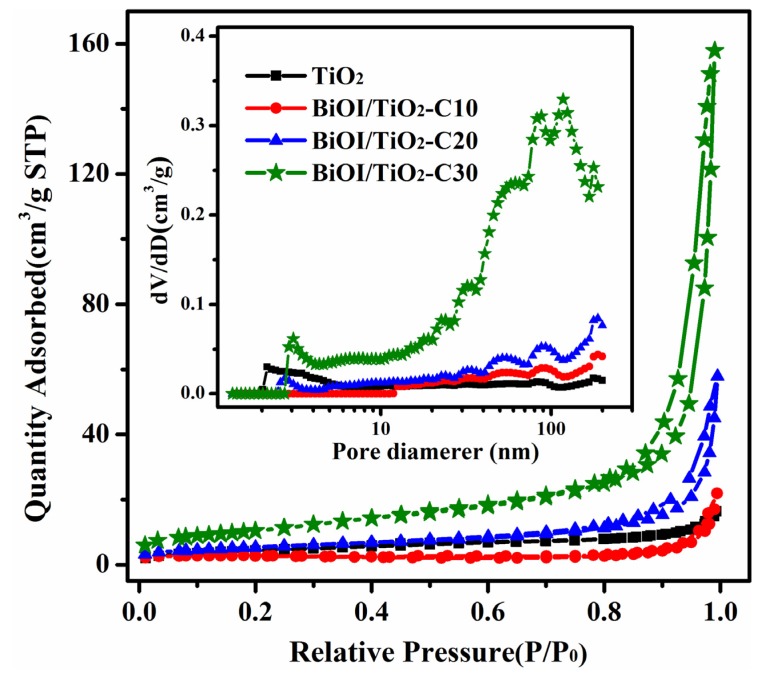
Typical N_2_ gas adsorption desorption isotherms of different samples and their corresponding pore-size distributions (inset).

All the samples have typical type-IV N_2_ adsorption-desorption isotherms with H1 hysteresis indicative of mesoporous structure (Figure 5). The curve of pure TiO_2_ nanofibers implies a meso- and macropore structure. As we know, the precursor nanofibers of electrospun TiO_2_ nanofibers consist of polymer and metal salt. During the calcination process, the decomposition of the polymer and metal salt can result in abundant hierarchical pores with a wide pore size distribution of more than 2 nm, as shown in Figure 5 inset. For BiOI/TiO_2_-C10, there is an obvious hysteresis loop in the large relative pressure range of 0.9–1.0 (P/P_0_), indicating the relatively large pore structure arising from the voids among the BiOI nanosheets on TiO_2_ nanofibers. The specific surface areas of these samples are shown in Table 1. It is also worth noting that the BiOI/TiO_2_-C10 exhibit lower specific surface areas than that of TiO_2_ nanofibers, which can be ascribed to the deposition of BiOI nanosheets blocking the original pores on TiO_2_. It can be clearly seen that the relative small pores (2–11 nm) are disappeared, which can be demonstrated by the pore size distribution of BiOI/TiO_2_-C10 in Figure 5 inset. Compared to BiOI/TiO_2_-C10, there is an obviously increased adsorption at high pressure with increased deposition of BiOI nanosheets on TiO_2_ nanofibers for BiOI/TiO_2_-C20 and BiOI/TiO_2_-C30, along with increased specific surface areas, indicating the more and more abundant porosity structures. It is accepted that the porosity is relative to the amount of BiOI nanosheets depositing on TiO_2_ nanofibers. Hence, the close arrangements of BiOI nanosheets on TiO_2_ nanofibers (see SEM and TEM images) have resulted in the hierarchical porosity with wide pore size distributions, which are further confirmed by the corresponding pore size distributions in the inset of Figure 5. These results suggest that the BiOI/TiO_2_ nanofibers with abundant porosity and large specific surface areas will increase the assessable surface areas of the catalyst with dye solution to achieve good photocatalytic activity. Particularly, the large amount of BiOI nanosheets depositing on TiO_2_ nanofibers without independent nucleation will benefit the formation of more p-n heterojunctions as well as rapid charge transfer during the photocatalysis.

**Table 1:** SILAR cycles, BET specific surface area and photocatalysis reaction rates of different samples.

Samples	Cycles	BET Specific Surface Area (m^2^/g)	K_app_ (h^−1^)
TiO_2_ NFs	0	15.88	0.000 ± 0.000
BiOI/TiO_2_-C10	10	7.27	0.141 ± 0.002
BiOI/TiO_2_-C20	20	19.19	0.197 ± 0.009
BiOI/TiO_2_-C30	30	38.44	0.724 ± 0.095
M-BT (Bi:Ti = 0.4:1)	-	-	0.267 ± 0.024

### 2.5. Optical Properties

**Figure 6:**
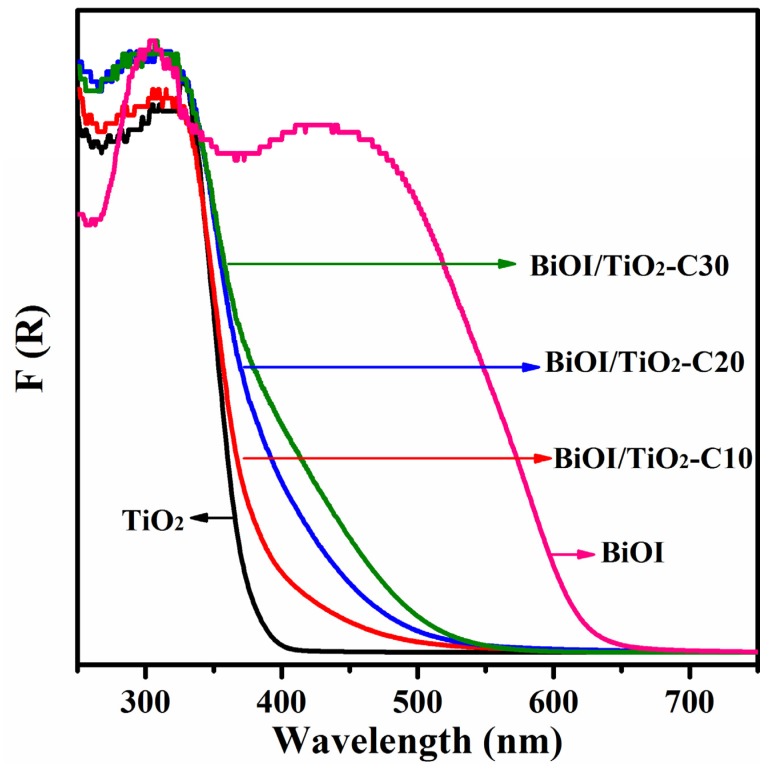
UV-vis absorption spectra of different samples.

Figure 6 shows the UV-vis absorption spectra of TiO_2_, BiOI/TiO_2_-C10, BiOI/TiO_2_-C20, BiOI/TiO_2_-C30 and BiOI converted from corresponding diffuse reflectance spectra by means of the Kubelka–Munk function [28]:

(1) *F(R) = (1 − R)^2^/2R = α/S*

(2) *R = R_Sample_/R_BaSO4_*

where *R*, *α*, and *S* are the reflectance, absorption coefficient and scattering coefficient, respectively. It can be seen that TiO_2_ exhibited a typical absorption characteristic of the wide band gap semiconductor with an edge about 380 nm, while pure BiOI with a strong absorption at about 630 nm in the visible light region, indicates that it is a narrow band gap semiconductor according to the equation E_g_ = 1240/λ, where E_g_ is the band gap (eV) and λ (nm) is the wavelength of the absorption edge in the spectrum. The band gap of TiO_2_ and BiOI are estimated to be 3.2 eV and 1.9 eV, respectively. It is noted that the absorption edge of p-BiOI/n-TiO_2_ NFs show significant red-shift from 393 to 500 nm with the increased amount of BiOI in the composite nanofibers. Based on the above, the increased amount of BiOI in p-BiOI/n-TiO_2_ NFs extends light absorbing range, which is the precondition of effective photocatalytic activity.

### 2.6. Photocatalytic Properties

**Figure 7:**
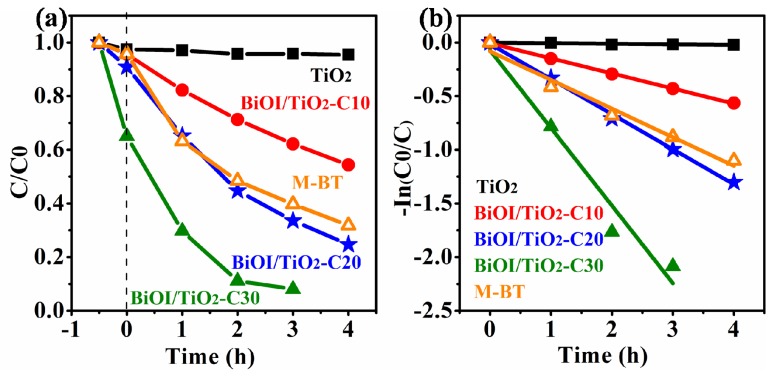
(**a**) Degradation curves of MO under visible light irradiation; and (**b**) the apparent first-order kinetics fitting over different samples.

Figure 7a shows the photocatalytic activities of TiO_2_ NFs, BiOI/TiO_2_-C10, BiOI/TiO_2_-C20, BiOI/TiO_2_-C30 and the mechanical mixture of BiOI and TiO_2_ (M-BT, the molar ratio of Bi:Ti = 0.4:1 based on energy dispersive X-ray (EDX) analysis in Figure S1) on the degradation of methyl orange (MO) under visible-light irradiation (≥420 nm). Before irradiation, the adsorption-desorption equilibrium of MO in the dark is established within 30 min over different samples. The time-dependent absorbance spectra of different samples are shown in Figure S1. The adsorption of BiOI/TiO_2_-C30 increases significantly compared to other samples, which might be attributed to the high specific surface area. After 3 h irradiation, the photodegradation efficiencies of MO for BiOI/TiO_2_-C30 are about 92%, in comparison to 60%, 66%, 38% and almost none for M-BT, BiOI/TiO_2_-C20, BiOI/TiO_2_-C10 and TiO_2_ nanofibers, respectively. In Figure 7b, the kinetic linear fitting curves over different photocatalysts show that the photocatalytic degradation of MO followed a Langmuir-Hinshelwood apparent first-order kinetics model:

(3) In *C*/*C_0_* = − *kKt* = − *k*_app_*t*

where *C_0_* is the initial concentration (mg/L) of the reactant; *C* is the concentration (mg/L); *t* is the visible-light irradiation time; *k* is the reaction rate constant (mg/(L·min)); and *K* is the adsorption coefficient of the reactant (L/mg); *k*_app_ is the apparent first-order rate constant (min^−1^). The *k*_app_ of different samples are shown in Table 1. It is indicated that the photocatalytic activities is in the order of BiOI/TiO_2_-C30 > BiOI/TiO_2_-C20 > M-BT > BiOI/TiO_2_-C10 > TiO_2_. The above illuminates that the construction of p-n heterojunctions can effectively enhance the photocatalytic properties. Furthermore, the increased of the specific surface area and the amount of p-n heterojuctions obviously enhance the photocatalytic activity. Furthermore, the photocatalysis under UV-light irradiation (Figure S2) also demonstrates the above point.

**Scheme 1:**
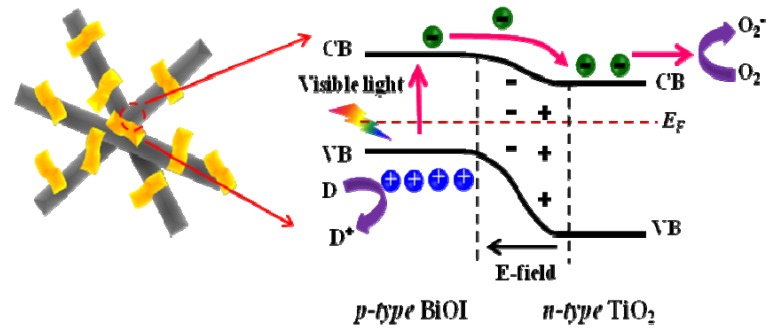
Possible photocatalytic reactions of p-BiOI/n-TiO_2_ NFs heterojunctions.

**Figure 8:**
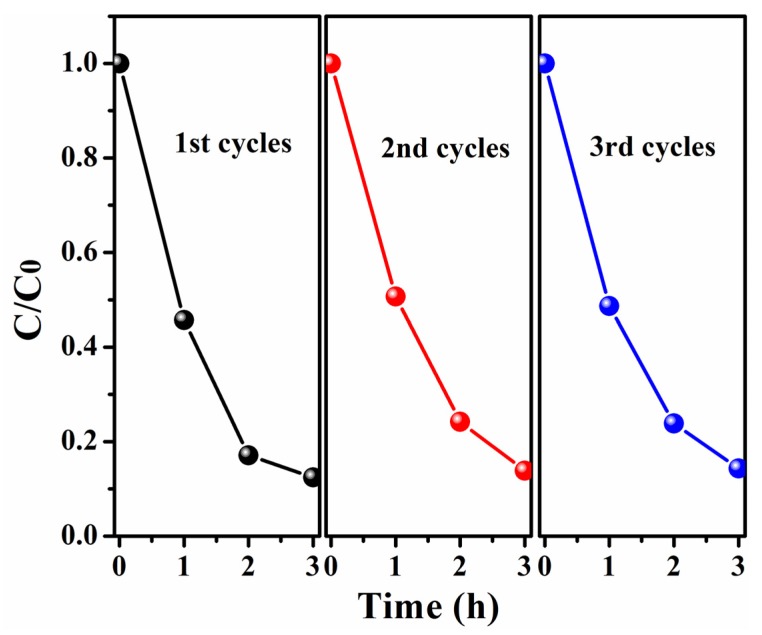
Photocatalysis tests of BiOI/TiO_2_-C30 for three cycles.

To understand the photocatalytic properties of p-BiOI/n-TiO_2_ NFs, a schematic diagram is proposed (Scheme 1). When p-type BiOI contacts n-type TiO_2_, the diffusion of electrons and holes create an inner electric field where a space-charge region is formed at the interfaces of p-n heterojunction. Under visible-light irradiation, the photogenerated electrons transfer from the conduction band of BiOI to that of TiO_2_, while the photogenerated holes stay at the valence band of BiOI. The recombination of photogenerated charge carrier is inhibited greatly in the heterojunctions of p-BiOI/n-TiO_2_ NFs. Thus, the photogenerated electrons and holes can effectively take part in the photodegradation of MO under visible light. On the other hand, the nanofiber structures of TiO_2_ can prevent the agglomeration of BiOI nanosheets and facilitate the transfer of the dye molecules during photocatalytic process. Moreover, the exposed surface of BiOI is mainly {001} facet, which is very active for photocatalytic reactions under visible-light irradiation [32]. Thus, the nanosheet structure of BiOI might also improve the surface reaction rates and contribute to the photocatalysis. It should be noted that the p-BiOI/n-TiO_2_ NFs can be easily separated from an aqueous suspension for reuse due to their one-dimensional nanofibrous morphology. As shown in Figure 8, the photodegradation of MO on the p-BiOI/n-TiO_2_ NFs was reused three times. Each experiment was carried out under identical conditions. Clearly, the photocatalytic activity of p-BiOI/n-TiO_2_ NFs remains almost unchanged after three-cycles, suggesting that the BiOI/TiO_2_ NFs have good stability and recycling properties.

## 3. Experimental

**Scheme 2:**
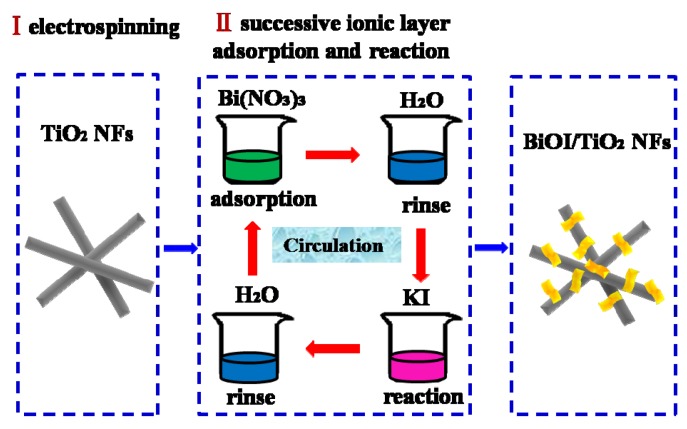
Schematic illustration for the preparation of p-BiOI/n-TiO_2_ NFs heterojunctions.

The synthesized process of samples is presented in Scheme 2.

### 3.1. Fabrication of TiO_2_ Nanofibers

Firstly, 1.6 g Poly(vinyl pyrrolidone) powder (PVP, Mw = 1,300,000) was added to a mixture of 20 mL absolute ethanol and 2 mL acetic acid in a Erlenmeyer flask. The obtained solution was stirred for 2 h to generate a homogeneous solution. Then, 2.0 mL Ti(OC_4_H_9_)_4_ was added to the solution, the mixture was magnetically stirred for another 10 h at room temperature to make electrospinning precursor solution. Subsequently, the above precursor solutions were drawn into a hypodermic syringe with a needle tip. Then, a high voltage source was connected to the needle tip while a sheet of aluminum foil was employed as the collector. The voltage between the needle tip and collector was set at 10 kV, and the distance was 15 cm. The as-collected nanofibers were calcined at a rate of 25 °C/h and remained for 2 h at 520 °C to obtain TiO_2_ NFs.

### 3.2. Fabrication of BiOI/TiO_2_ Nanofibers

The p-BiOI/n-TiO_2_ NFs were synthesized through the SILAR process. Typically, 0.25 mM Bi(NO_3_)3·5H_2_O solutions were prepared with deionized water as solution A, and equivalent concentration of KI solution were prepared as solution B. The TiO_2_ nanofibers were first immersed into solution A for 2 min, rinsed with deionized water, and then immersed into solution B for 2 min, rinsing likewise. The four-step procedure forms one cycle and the BiOI would increase by repeating the cycles. A series of samples, with different cycles of 10, 20 and 30 were prepared and denoted as BiOI/TiO_2_-C10, BiOI/TiO_2_-C20 and BiOI/TiO_2_-C30. After that, the samples were thoroughly rinsed with deionized water and allowed to dry at 60 °C overnight. All the samples are listed in Table 1. Pure BiOI nanosheets were prepared by mixing solution A and B, then rinsed and dried.

### 3.3. Characterizations

Sanning electron microscopy (SEM, Quanta 250 FEG, FEI, Hillsboro, OR, USA) and high-resolution transmission electron microscopy (HRTEM; JEOL JEM-2100, JEOL, Tokyo, Japan) were used to characterize the morphologies of the products. The X-ray diffraction (XRD) measurements were carried out using a D/max 2500 XRD spectrometer (Rigaku, Tokyo, Japan) with a Cu Kα line of 0.1541 nm. The X-ray photoelectron spectroscopy (XPS) was performed on a VG-ESCALAB LKII instrument (VG, Waltham, UK) with Mg KαADES (hυ = 1253.6 eV) source at a residual gas pressure of below 10^−8^ Pa. The specific surface area of the samples were measured with a Micromeritics ASAP 2010 instrument (Micromeritics, Norcross, GA, USA) and analyzed by the Brunauer-Emmett-Teller (BET) method. The UV-vis diffuse reflectance spectra were measured at room temperature with a UH4150 spectrophotometer (Hitachi, Tokyo, Japan).

### 3.4. Photocatalytic Tests

A 150 W xenon lamp with a cut off filter (≥420 nm) was used as the visible light source for photocatalysis. Using MO as model pollutants, photocatalyst (0.1 g) was suspended in MO solution (100 mL, 10 mg/L) with stirring. The solution was kept in the dark for 30 min to reach adsorption-desorption equilibrium between the organic molecules and the photocatalyst surface. Then, 4 mL reacted solutions in series were taken out and analyzed every 1 h. The concentrations of MO in the reacting solutions were analyzed by a Cary 500 UV-vis-NIR spectrophotometer (Varian, Palo Alto, CA, USA) at 464 nm.

## 4. Conclusions

In summary, using electrospinning technology and SILAR method, heterojunctions of p-BiOI/n-TiO_2_ NFs have been successfully fabricated. Due to the p-n heterojunction effects and large specific surface area, the BiOI/TiO_2_-C30 exhibits higher visible-light photocatalytic behavior in comparison with other samples for degradation of MO. Furthermore, the p-BiOI/n-TiO_2_ NFs can be easily recycled without a decrease of the photocatalytic activity because of their nanofibrous nonwoven web structure property. It is expected that the p-BiOI/n-TiO_2_ NFs with high photocatalytic activity will greatly promote their industrial application to eliminate the organic and inorganic pollutants from wastewater.

## Supplementary Materials

The following are available online at www.mdpi.com/1996-1944/9/2/90/s1.
