# Supplementary material for: Heterojunctions of p-BiOI Nanosheets/n-TiO2 Nanofibers: Preparation and Enhanced Visible-Light Photocatalytic Activity
Source: Materials (Basel). 2016 Jan 30;9(2):90. doi: 10.3390/ma9020090 (PMC5456490; doi:10.3390/ma9020090)
Supplement: Supplementary file 1 [file materials-09-00090-s001.pdf]

Supplementary Materials: Heterojunctions of p-BiOI Nanosheets/n-TiO_2 Nanofibers: Preparation and Enhanced Visible-Light Photocatalytic Activity

Kexin Wang, Changlu Shao, Xinghua Li, Fujun Miao, Na Lu and Yichun Liu

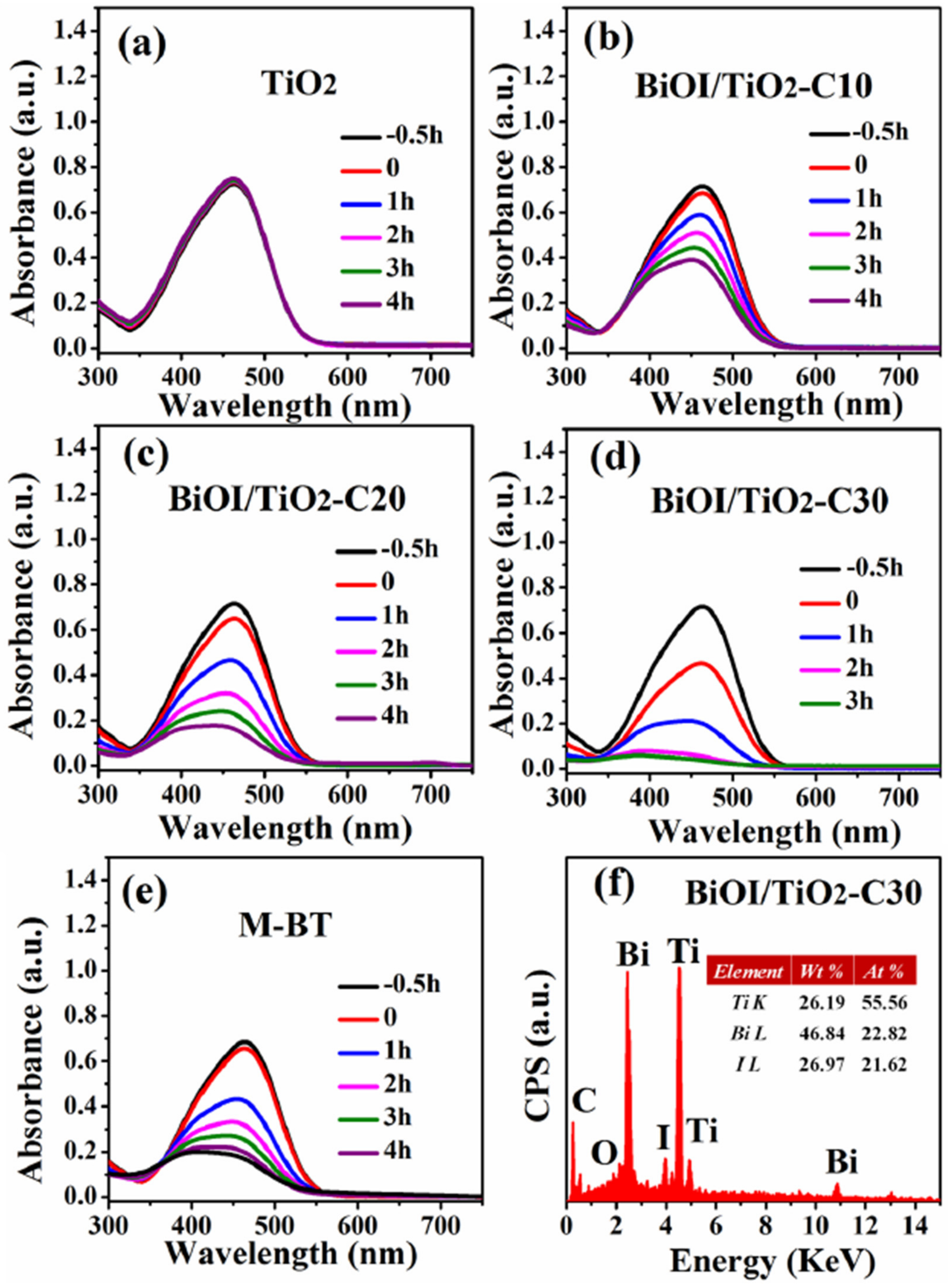

Figure S1. Time-dependent UV-vis absorbance spectra of the MO solution in the presence (**a**) TiO_2; (**b**) BiOI/TiO_2-C10; (**c**) BiOI/TiO_2-C20; (**d**) BiOI/TiO2-C30; (**e**) mechanical mixture of BiOI and TiO_2 (M-BT) under UV light irradiation; (**f**) EDX of BiOI/TiO_2-C30 (Bi:Ti = 0.4:1).

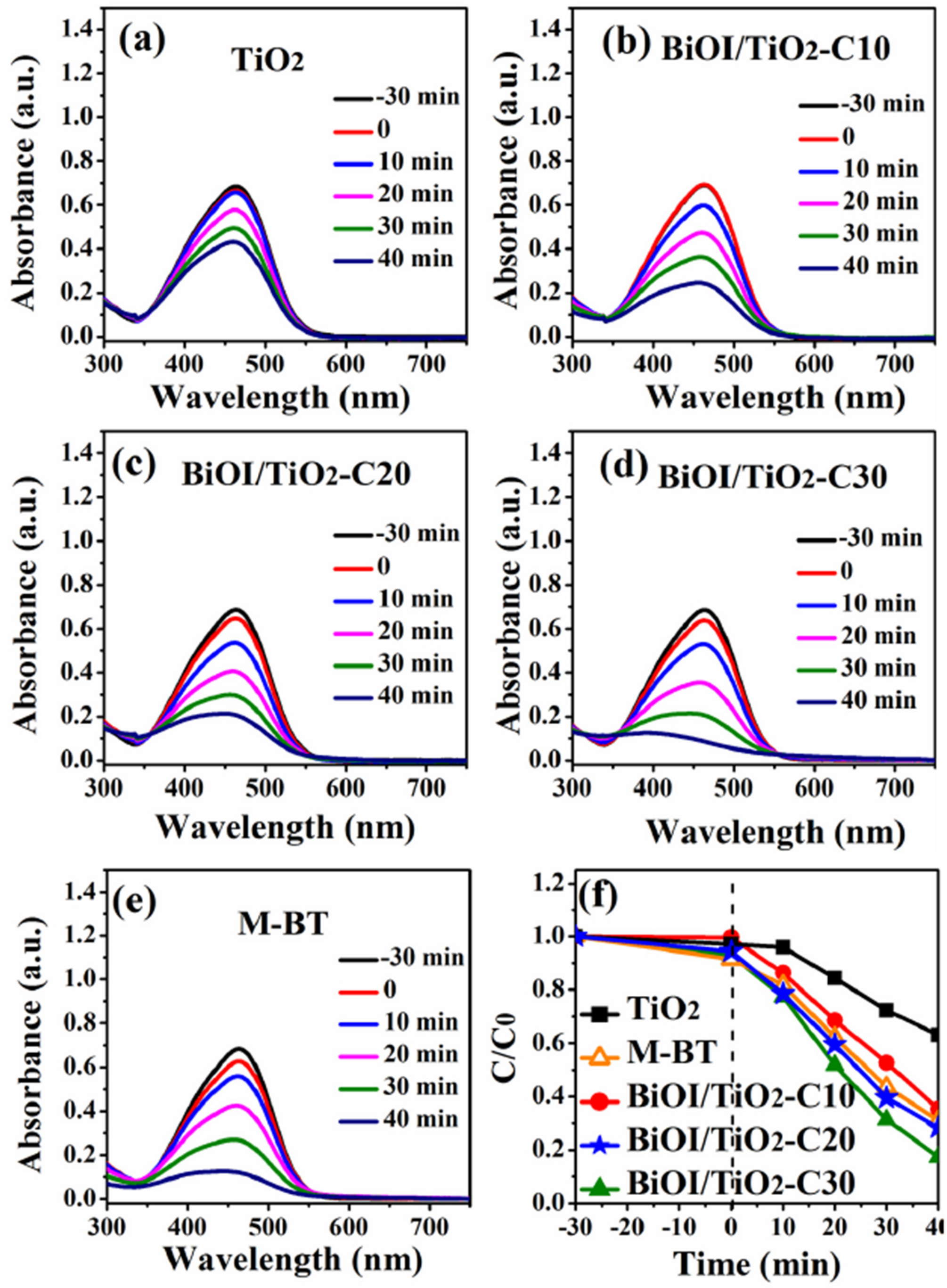

Figure S2. Time-dependent UV-vis absorbance spectra of the MO solution in the presence (**a**) TiO_2; (**b**) BiOI/TiO_2-C10; (**c**) BiOI/TiO_2-C20; (**d**) BiOI/TiO_2-C30; (**e**) mechanical mixture of BiOI and TiO_2 (M-BT); (**f**) degradation curves of MO under UV light irradiation.

Figure S2a–c shows the time-dependent UV-vis absorbance spectra of the MO solution in the presence TiO_2 NFs, BiOI/TiO_2-C10, BiOI/TiO_2-C20, BiOI/TiO_2-C30 and the mechanical mixture of BiOI and TiO_2 (M-BT, the molar ratio of Bi:Ti = 0.4:1 based on EDX analysis in Figure S1), respectively. The photocatalytic activities over different samples are showed in Figure S2d. Before irradiation, the adsorption-desorption equilibrium of MO in the dark is established within 30 min. After 40 min irradiation, the photodegradation efficiencies of MO for BiOI/TiO_2-C30 are about 93%, in comparison to 69%, 72%, 65% and 37% for M-BT, BiOI/TiO_2-C20, BiOI/TiO_2-C10 and TiO_2 nanofibers, respectively.
